# Impact of malt concentration in solid substrate on mycelial growth and network connectivity in *Ganoderma* species

**DOI:** 10.1038/s41598-023-48203-4

**Published:** 2023-11-29

**Authors:** Natalie Nussbaum, Tabea von Wyl, Antoni Gandia, Edwina Romanens, Patrick Alberto Rühs, Peter Fischer

**Affiliations:** 1 ETH Zürich, Institute of Food, Nutrition and Health, 8092 Zurich, Switzerland; 2 Planted Foods AG, Kemptpark 32, 8310 Kemptthal, Switzerland; 3 IBMCP (UPV-CSIC), Institute for Plant Molecular and Cell Biology, 46011 Valencia, Spain

**Keywords:** Spectrophotometry, Bioinspired materials, Fungal ecology, Mechanical properties

## Abstract

With its distinctive material properties, fungal mycelium has emerged as an innovative material with a diverse array of applications across various industries. This study focuses on how the growth strategies of wood fungi adapt to nutrient availability. The effect of malt extract concentration in the growth medium on radial growth kinetics, morphology, mycelium network connectivity, and mechanical characteristics of mycelium from two *Ganoderma* species were investigated. While an evident pattern of radial growth rate enhancement with malt concentrations was not apparent, there was a discernible trend towards denser mycelium network characteristics as revealed by spectrophotometry. Increased malt extract contents corresponded to elevated optical density measurements and were visually confirmed by denser mycelium networks in photographic images. Investigating the mechanical characteristics of mycelium cultivated on varying solid substrate concentrations, the Young’s modulus exhibited a substantial difference between mycelium grown on 5 wt% malt substrate and samples cultivated on 2 wt% and 0.4 wt% malt substrates. The obtained results represent a new understanding of how malt availability influences mycelial growth of two *Ganoderma* species, a crucial insight for potentially refining mycelium cultivation across diverse applications, including meat alternatives, smart building materials, and alternative leather.

## Introduction

Fungal mycelium is a promising material with applications in various industries, such as packaging, smart building materials, food, and leather alternatives^1–5^. In the food industry, mycelium is particularly attractive for meat alternatives, as it can convey meat-like texture and umami flavor, which is often associated with meat products^6,7^. A few examples of mycelium-based foods are those currently produced and commercialized by companies such as Marlow Foods Ltd. (North Yorkshire, UK), Meati Foods (Colorado, US), The Better Meat Co. (California, US), Mycorena (Gothenburg, SE), and Nature’s Fynd (Illinois, US), mainly using liquid-state fermentation methods from which wet fungal biomass is obtained. Such biomass, also known as mycoprotein^8,9^, is post-processed and transformed into different products such as minced meat replacements, cold cuts, and whole cuts recreating chicken breast, bacon, and steaks. On the other hand, the company MyForest Foods Co. (New York, US) is well known for using a proprietary solid-state fermentation technology that renders foamy slabs of textured mycelium^10–12^.

To optimize the application of fungal material, it is important to understand how to guide and control mycelial growth, as well as how to employ the broad biochemical toolbox of fungal growth structure. In particular, for the use of fungi as meat alternatives or even as leather-like materials, mechanical characteristics, and texture are of great interest.

The mycelium of Basidiomycota first grows isotropically, until forming fractal tree-like colonies, leading to a randomly interconnected network^13^. Basidiomycota are a phylum of fungi that produce spores through specialized structures called basidia and include mushroom-forming fungi^14^. The relevation of distinct growth strategies in response to varying nutrient availability in some Basidiomycetes adds a layer of complexity to our understanding of fungal biology. When exposed to an abundance of easily accessible nutrients, the mycelium can employ the *phalanx* strategy, where the organism progresses steadily in a unified front, producing thick and highly branched hyphal mats. Conversely, in environments characterized by limited local nutrition, the mycelial growth shifts to an exploration form known as *guerilla*. In this mode, the organism demonstrates an opportunistic nature, extending their hyphae far and wide with reduced branching^15,16^. The motivation behind studying these interesting fungal growth patterns stems from the field of plant ecology, particularly in investigating how clonal plants grow^17^. The idea of *phalanx/guerilla* growth forms in clonal plants aligns with describing fungal mycelial growth patterns. The mechanical and spatial properties of the substrate, which eventually define the growth window are largely uncharted. As fungi follow the path of least resistance toward nutrients and oxygen, directed growth could be achieved by creating stimuli-triggering pathways for the fungi^18–20^. Furthermore, in recent years, research on the mechanical properties of mycelium materials focused mainly on the effect of fungal phenotype, cultivation, and substrate^2,21,22^. However, the sample preparation prior to testing also affects the mechanical stability of mycelium materials^23^.

The aim of the present work is to investigate the mycelial network of two *Ganoderma* species and the effect of network density on mechanical characteristics. Mycelium growth kinetics and network density were measured in relation to nutrient supply using radial growth measurements, spectrophotometry, and tensile testing. Tensile testing is a mechanical test, where a material is stretched to its breaking point to understand its response to applied forces. During the test, a sample of the material is pulled in opposite directions, subjecting it to tension. By measuring the resulting stress (force per unit area) and strain (deformation), one can obtain Young’s modulus, which is the slope from the stress-strain curve, quantifying the material’s ability to resist deformation when subjected to tension^24^. A higher Young’s modulus of mycelium has been linked to increased density of the material in terms of weight-to-volume ratio^22^. In the present study, density is also linked to mycelial connectiveness and branching. Within that perspective, a highly dense mycelial network describes the opposing phenomenon of hyphal aggregation. Thus, this study aims to display the use of nutrient availability to direct mycelium growth and mechanical characterization as well as to point out the importance of growth conditions and sample preparation to compare obtained Young’s moduli.

## Results and discussion

### Screening of species

**Figure 1:**
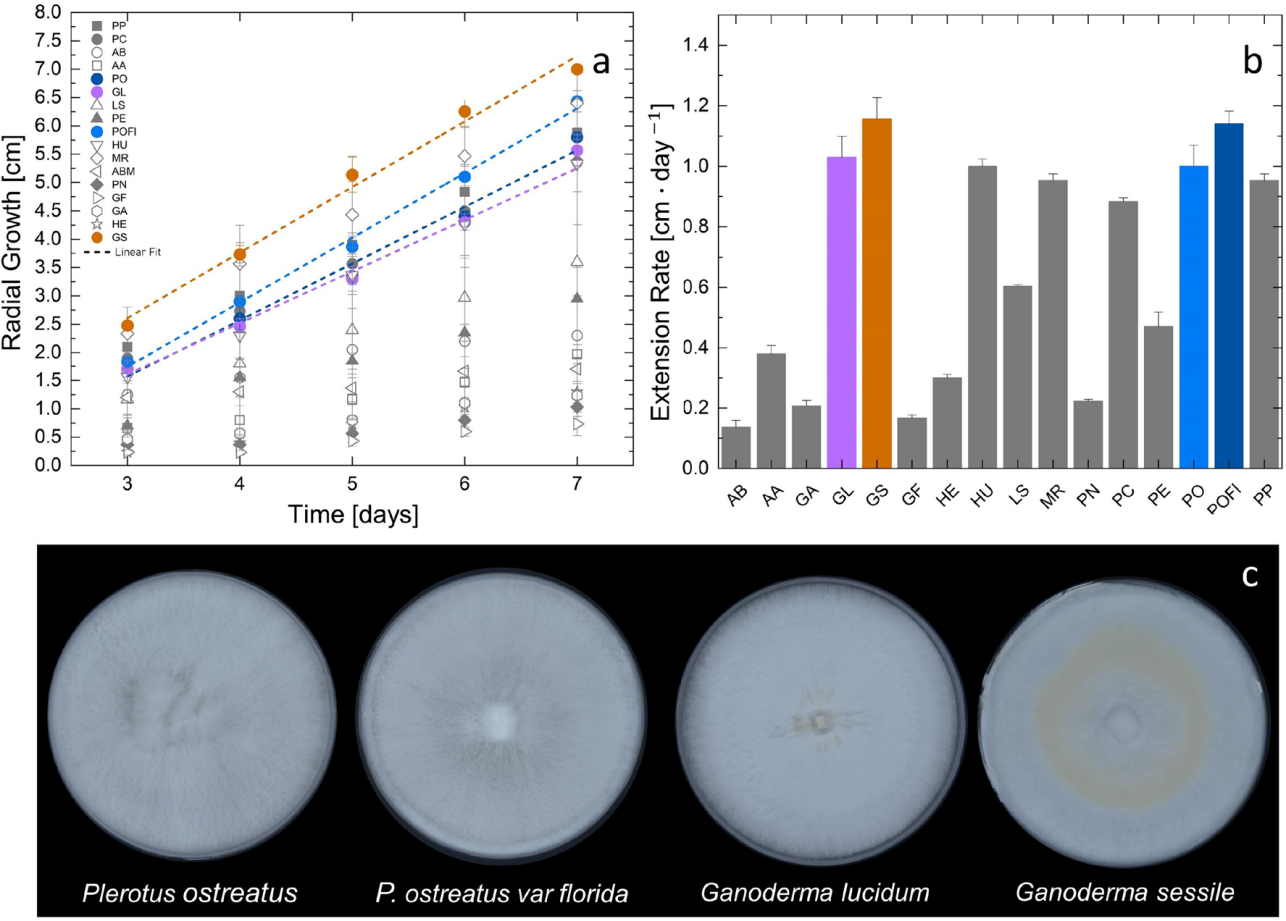
Growth kinetics of various wood fungi species are shown as (**a**) radial growth over time (days) and as (**b**) radial extension via fitting the growth curves seen in (**a**) (n = 3). Note: Linear fits with adjusted R squares> 0.99 were considered. (**c**) Pictures of mycelial morphology of analyzed *Pleurotus* spp. and *Ganoderma* spp. after 7 days incubation at $30\,\,^{\circ }\text {C}$ and 80% RH.

The results of the screening process of various fungal species are summarized in Fig. 1. Fungal species and their abbreviations are listed in the Supplementary Information (Table S1). To perform an extensive screening of different fungal species, growth was tracked starting on the third day until the plate was completely overrun (Fig. 1a). Linear radial growth of Basidiomycetes mycelium was confirmed after day 3 of cultivation in a preliminary experiment (Fig. S2). On standard 2 wt% malt agar (SMA) media *Ganoderma sessile*, *Pleurotus ostreatus* var *florida*, *Ganoderma lucidum*, and *Pleurotus ostreatus* proliferated fastest among the 18 analyzed fungal species with radial growth rates ranging between 1 and 1.16 cm/day. In comparison, the slowest species studied, *Agaricus blazei*, evolved at a speed of 0.14 cm/day (Fig. 1b). Images of the Petri dishes were taken after 7 days of incubation (Fig. 1c). Ganoderma species showed the most densely packed and regularly arranged radial growth patterns in morphological images.

According to the results from the screening, *G. sessile* was further selected for this study because of the observed promising mycelial growth features, which have also been noticed in the production of bio-composite materials and fungal skins^25,26^. In addition, *G. lucidum* was included as a recognized edible species within the same genus^27^ (see Supporting Figure S1) and given its widespread presence in fungal biomaterial-related literature^2,21,28^, which allows for a side-to-side comparison of the findings aiming toward food-focused applications.

To outline the extent of the influence of nutrient concentration on mycelial growth behavior, we analyzed the following aspects: (1) the growth kinetics and mycelium morphology, (2) the mycelium density and biomass, and (3) the mechanical behavior of the mycelium.

### Growth kinetics and morphology

**Figure 2:**
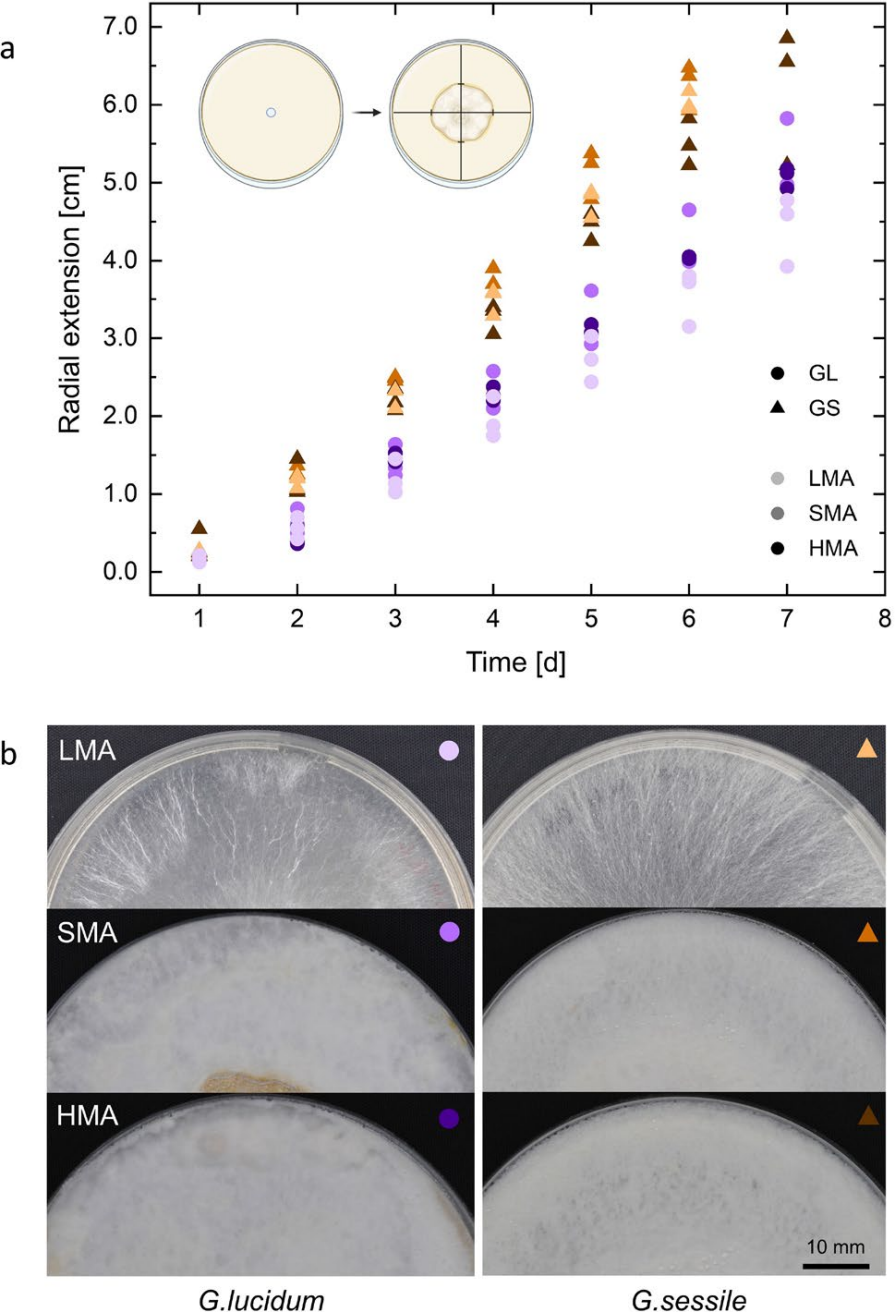
(**a**) Growth kinetics of *Ganoderma lucidum* (GL) and *Ganoderma sessile* (GS) grown on LMA, SMA, and HMA over 7 days, measured via monitoring daily radial growth. Data points of 3 biological replicates each are shown. Note: Some data points for day 7 are not shown as the Petri dishes were overgrown to the edge. (**b**) Representative morphology of examined plates grown for 7 days.

The growth speed of the two species was tested on three agar substrates with distinct malt concentrations: low malt agar with 0.4 wt% malt extract (LMA), standard malt agar with 2 wt% malt extract (SMA), and high malt agar with 5 wt% malt extract (HMA). We found that the tested species exhibited considerable variations (Fig. 2a). In both species, there was a noticeable trend toward slightly faster mycelial growth on the 2% malt substrate (SMA) compared to HMA and LMA, although the results did not exhibit substantial differences. This is contrary to our expectations and the *phalanx/guerilla* theory, where the fungal species grow slower in the high-nutrient environment and faster when nutrient availability is low. To investigate the effect of increased nutrients in the substrate on growth kinetics, additional tests with malt concentrations ranging from 2 to 8 wt% were performed (see Supporting Figure S4). Interestingly, both species present the recurring trend of experiencing their most rapid growth on 2 wt% ME, even when compared to higher ME concentrations up to 8%. Furthermore, comparing the mean (n = 3) growth of the two fungi on SMA, *G. sessile* exhibited faster linear growth (1.24 cm/day) than *G. lucidum* (0.94 cm/day).

To allow comparison between the various substrates, images of mycelium of the same age and incubated under the same conditions were taken on the respective borders of the colony (Fig. 2b). The images indicate variations in growth depending on the substrate used. In particular, when grown on LMA, the mycelial network is less branched, and individual strands are agglomerated.

For all tested media, *G. sessile* developed faster in radial growth and denser in terms of biomass per area than *G. lucidum*. Moreover, both of the studied species exhibited the capacity to adapt their growth patterns in response to the malt extract concentration available. However, the relationship between growth rate and malt extract concentration differed between the two species: *G. lucidum* demonstrated the fastest growth on SMA, slightly slower on HMA, and the slowest growth on LMA. Conversely, *G. sessile* exhibited the quickest development on SMA, slower growth on LMA, and the slowest growth on HMA. The fast and aggressive growth of *G. sessile* has also been observed in previous studies, where *G. sessile* shows a great fitness growing across different wood types^29,30^. Moreover, *G. sessile* prefers to infect living trees, whereas *G. lucidum* grows mostly saprotrophically in nature^31^. While this behavior seems to be related to the preference for simple or complex carbohydrates in the substrate rather than their concentration, these apparently differential metabolisms within the genus offer a promising foundation for future research on various kinds of substrates in vitro. We want to note that in this study, two different species were studied, and thus, the observed differences could also be species-dependent.

We have seen that there is an optimum malt concentration in terms of the growth kinetics of the two studied *Ganoderma* species. Nevertheless, morphological images of *G. lucidum* demonstrate that the mycelium operates differently in nutrient-poor media, with a less branching network and more agglomerated individual strands. This finding is supported by visual observations of hyphal strands congregating to extend over nutrient-deficient areas, which corresponds to similar findings in literature^15,32,33^. The formation of hyphal aggregates (cords) in a nutrient-depleted environment is likely due to higher transport capacity over longer distances^33^. Denser mycelium formations on nutrient-rich media are in line with *phalanx/guerilla* exploitation strategies of fungi. Regarding kinetics, the *phalanx/guerilla* exploitation theory further suggests variable growth kinetics according to nutrient availability. However, based on our data, we are unable to affirm a distinct pattern of enhanced growth kinetics with elevated malt concentrations in both strains. Rather, growth reached a plateau at a certain malt concentration and could not be increased by adding more malt extract to the substrate. In this experiment, malt extract served as the carbon source. As malt extract contains non-carbohydrate components^34^, the described effects could also imply stochiometric limitation of growth, which has been observed with various nutrients in yeast and bacterial strains^35^. We observed substantial growth effects with malt, a widely used carbon source in fungal cultivation^5,20,28,30^, prompting us to continue our research using malt as a carbohydrate source. Notably, should one desire to attribute the impact solely to the carbohydrate content, we suggest using glucose or sucrose as the carbon source, for instance.

### Mycelium density

**Figure 3:**
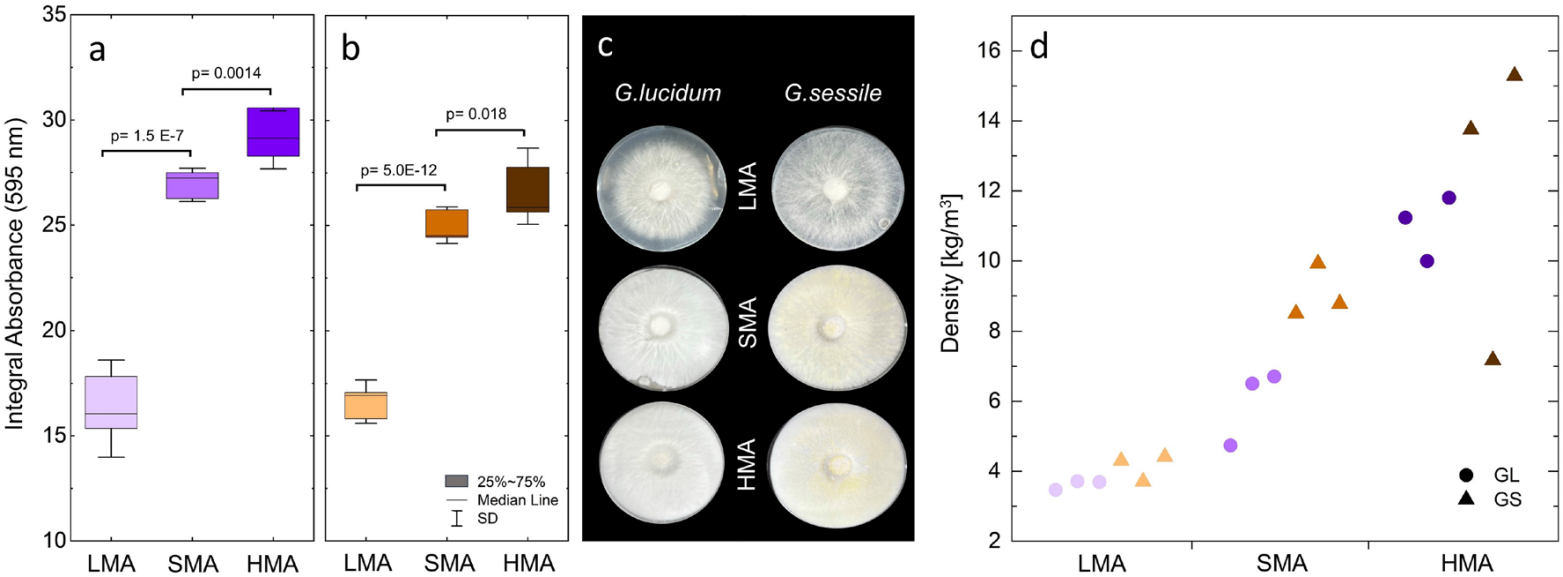
Mycelium characterization via integrating OD values after 7 days of growth on various substrates via spectrophotometry (**a**) *Ganoderma lucidum* and (**b**) *Ganoderma sessile*. Bars representing standard errors are displayed for each mean. Photographic images of mycelium morphology (**c**) and mycelium density assessed via fungal mat weight estimation (**d**).

Mycelium density was assessed via optical density (OD) measurements. The signals from mycelium colonies formed in one microplate were distributed with higher OD in the center of the colony and lower ODs near the borders of the colonies (Fig. S5). It is, therefore, reasonable to assume that OD595 measurements of mycelium acquired with this technique mirror the kinetics of fungal growth^36^. To investigate the effect of nutrient availability, the OD signal as a function of time for the mycelium growing on the three distinct substrates (LMA, SMA, and HMA) was analyzed. The absorbance values in Fig. S6 relate to the mycelium biomass in one well from the inoculation point to the edge as described in the “Methods” section. There was a visible trend in mycelium density after 7 days of incubation between the different growth substrates used (Fig. 3a and b). In both species, higher malt extract contents were associated with higher mycelium density reflected in higher optical density measurements. This finding is supported by photographic images of the respective samples at that time (Fig. 3c), which depict denser mycelium networks in HMA and SMA compared to LMA. Even if no difference in morphology between SMA and HMA is visible to the eye, growing mycelium on SMA results in a lower mycelium density according to OD measurements.

Additionally, density values derived from fungal mat weight estimation were analyzed (Fig. 3d). For *G. lucidum*, biomass was significantly increased with nutrient-rich growth media from low- to standard and high malt substrate. For *G. sessile*, this effect is only visible between LMA and SMA. The increase in biomass of mycelium grown on substrates with higher malt concentrations confirms the results we obtained via OD measurements.

A trend of increased absorbance and biomass was observed in both species when the substrate contained higher amounts of malt extract. Thus, for future studies, it will be useful to know that while 0.4 wt% ME is too low to optimize growth speed, adding ME concentrations > 2 wt% will not necessarily lead to faster mycelium proliferation. Furthermore, considering material applications, it is worth noting that dense mycelial growth can contribute to stronger and more cohesive structures^37^. Absorbance measurements indicate that *G. sessile* and *G. lucidum* exhibit comparable mycelium network connectivity.

Most of mycelium density data of Basidiomycota in the literature is obtained via the ratio of biomass per unit of area/mass^22,37–39^ and absorbance in the context of fungi to measure biomass accumulation^36^. Herein, we show that absorbance (optical density, OD) at 595 nm wavelength can be used to assess mycelium network density. We observed a trend of denser mycelial growth with increasing malt extract concentration. Data collected with absorbance of mycelium on solid medium were in line with biomass formation as mycelium mats and were also confirmed by our visual observations on solid medium. Thus, we confirm that photometric measurement of fungal mycelium is a useful tool to quantify biomass accumulation and characterize fungal growth behavior and density. Higher absorbance measurements of mycelium with increasing nutrient availability are consistent with a prior study of filamentous fungi via spectrophotometry, where maximal biomass correlated with nutrient concentration^36^. Furthermore, an absorbance plateau with increased sugar concentration in the substrate is already indicated by the drop in absorbance increase from SMA to HMA. Additionally, the increase in biomass via weight with more abundant nutrients agrees with earlier research, where the dry weight of *G. applanatum* mycelia was increased with higher glucose concentrations^40^.

According to recent research on fungal mycelial growth on the hyphal scale, the original phalanx/guerrilla theory remains valid in terms of fungi exploration in space, but with some refinements. Aleklett et al.^20^ studied fungal growth characteristics more closely and discovered that the theory needs to be updated if one takes into account factors such as branching frequency, growth speed, mycelial expansion patterns, foraging range, and mycelial density. Here, we examined the network density and were able to demonstrate how both *G. lucidum* and *G. sessile* could alternate between the two strategies.

To assess mycelial growth behavior and density in future analyses, it may still be necessary to employ additional methods, such as radial growth and mycelial mat weighing, due to the limitations posed by the small sample size of photometric measurements on well plates.

### Mechanical characterization

**Figure 4:**
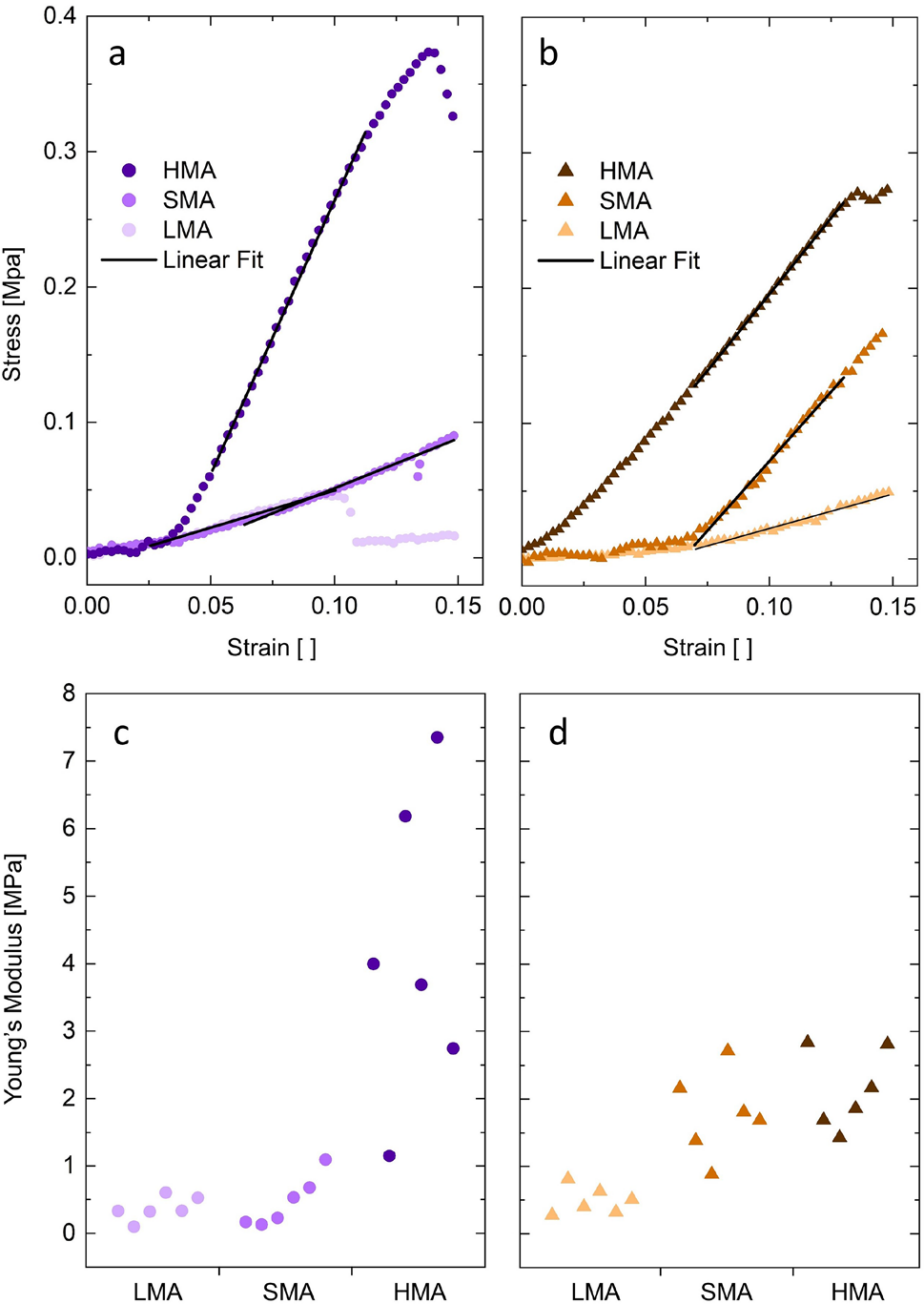
Mechanical characterization of mycelium material. Representative stress-strain curves from the mechanical testing used to calculate Young moduli E are shown in (**a**) for *G.lucidum* and (**b**) for *G.sessile*, including linear fits. Obtained Young Moduli E of 6 biological replicates for each condition are depicted in (**c**) for *G.lucidum* and (**d**) for *G.sessile*.

For mechanical characterization, mycelium stripes were tested after carefully harvesting them from solid substrate after 7 days of growth. Representative stress-strain curves for each growth condition are shown in Fig. 4a and b. The mycelium grown on the different tested substrates showed distinct behavior when exposed to tensile stress. The stress-strain curves display a predominantly linear trend in all measurements, following an initial lag phase observed in some samples. This observation implies that the sample may not have been fully loaded upon mounting (Fig. 4a and b). Young’s moduli were determined exclusively by considering the linear segment of the stress-strain curve. In Fig. 4c and d, obtained Young moduli of 6 biological replicates are shown for each condition. The Young’s moduli of the *G. lucidum* mycelial network formed on the HMA substrate was substantially higher (4.19 MPa) than that of the samples grown on SMA and LMA, while there was no big difference in Young’s modulus between SMA and LMA with mean E values of 0.47 MPa (SMA) and 0.37 MPa (LMA) (Fig. 4c). For *G. sessile*, the mean moduli were 0.49 (LMA), 1.77 (SMA), and 2.14 (HMA) (Fig. 4d). Thus, the effect of a more rigid mycelial network on HMA is more pronounced in *G. lucidum* than in *G. sessile*. A discernible variability in E was observed, and we postulate that this variation stems from the inherent nature of biological samples, where consistent growth cannot always be anticipated. Furthermore, the tedious preparation and mounting of substrates may contribute to a portion of the observed variability. Nevertheless, in both species, there is an observable pattern of E rising as the malt concentration in the substrate increases.

The mechanical measurements via tensile tests demonstrate stronger material characteristics of mycelium grown on malt-rich substrate. These results agree with the hypothesis of a more branched, denser mycelium network formation on high-sugar substrates. Assuming that substrates richer in sugars will lead to higher network density within mycelium, stronger elastic behavior is expected^41^. These results are supported by a study by Islam et al.^6^, where a higher Young’s modulus corresponds to denser mycelium materials. Furthermore, we observed a trend of a higher mean E value of the mycelium on HMA for *G. lucidum* compared to *G. sessile*. The findings of our absorbance research indicate that *G. lucidum* grows in higher mycelium density on HMA than *G. sessile*, which is consistent with the trend toward higher measured stiffness when grown on HMA. However, more strains of the same species would need to be examined to rule out the possibility that this observation is strain-dependent.

In contrast to our findings, other mechanical studies of mycelium materials have shown higher ductile behavior on carbon-rich substrates^21^. However, it is worth mentioning that in the referred study, mono- and polysaccharides were compared, and thus, the availability of sugar molecules was assessed rather than the concentration. Furthermore, cell wall composition and phenotype-related features offer an explanation for differences in the mechanical characteristics of mycelium of different fungal species^23^. The results from a few studies indicate that there is a correlation between phylogenetic organization and mechanical characteristics^21,23^. For instance, Haneef et al.^21^ found that materials derived from *Pleurotus ostreatus* are stiffer than those from *G. lucidum*, which is attributed to the increased amount of polysaccharides in *P. ostreatus*-based materials.

The measured moduli of the two studied *Ganoderma* species were lower than the values found in the literature^2,21,22,38^. For instance, Haneef et al.^21^ found a Young’s modulus of 2.5 MPa for *G. lucidum* grown on PDB-cellulose. A comparison of Young’s moduli of mycelium materials determined by tensile testing is shown in Supporting Figure S7. Within the data available, moduli vary greatly and E values in this study are lower in comparison. As there are few data from the mechanical assessment of Basidiomycota available in the literature, it is challenging to compare the obtained Young’s moduli. Furthermore, the existing discrepancies likely arise from factors such as growth parameters (including incubation time), expertise, phylogenetic differences, and inconsistencies in sample preparation^23^. Numerous steps from substrate preparation, incubation to specimen preparation and handling for testing must be taken into consideration when comparing the elastic moduli of mycelium materials. An overview of the various processing steps is given in Supporting Information Figure S8. Nevertheless, while we provide a summary of the literature, it is essential to point out that an in-depth comparison would require employing the same incubation method and expertise.

## Conclusions

In conclusion, the growth kinetics of the studied *Ganoderma* species, *G. lucidum* and *G. sessile*, reveal their ability to adapt growth strategies based on nutrient availability. Mycelium network density increases with malt content in the substrate, impacting mechanical strength of the mycelium. Optical density analysis proves valuable for assessing mycelium network density, growth, and biomass accumulation. Finally, the mycelium shows stronger material characteristics on malt-richer substrate, indicating the dependence of mechanical strength on mycelium network density, even though this effect is more pronounced in *G. lucidum* than *G. sessile*. Considering the discrepancies in the literature on fungal material properties, we emphasize the importance of consistent measuring protocols when comparing species. Depending on the application, *G. lucidum* is recommended over *G. sessile*, particularly for food-related uses, considering the Novel Food Regulation (NFR) within the EU. However, the mycelium can only be commercialized as a dietary supplement for immune system support, not as a foodstuff or ingredient. In contrast, the mushroom or basidiocarp of *G. lucidum* has a history of pre-1997 use in the EU and is approved for consumption (see Supporting Information S1).

Understanding and manipulating fungal growth, particularly mycelium development, is essential for advancing research and fostering innovation in materials and food. Future studies exploring nutrient availability at different depths could benefit from a comprehensive approach, integrating techniques such as Confocal Microscopy, Fluorescence Recovery After Photo-bleaching (FRAP), and High-Performance Liquid Chromatography (HPLC) for a nuanced understanding of nutrient uptake dynamics. In addition to nutrient composition, researchers should investigate other substrate factors, including porosity, micro- and macro-architectures, and viscosity, to unveil their impact on mycelium growth. These factors play a crucial role in establishing the optimal three-dimensional environment for mycelial proliferation and texture development.

## Methods

### Materials

Table 1 depicts the fungal species and Table 2 depicts the raw materials used for the substrate in this study. Experiments were performed almost exclusively with two fungal species from the genus Ganoderma, *Ganoderma lucidum* and *Ganoderma sessile*. In one experiment, other fungal species were also included (see Table S1 for detailed information).

**Table 1:** Fungal species used in this study, including collection code and supplier.

Code	Taxonomic identification	Phylum	Collection code	Supplier
GL	*Ganoderma lucidum*	Basidiomycota	MG11500	Mycogenetics, Germany
GS	*Ganoderma sessile*	Basidiomycota	95-19	MOGU S.r.l., Italy

**Table 2:** Raw material, including the supplier, used for substrate preparation.

Material	Supplier
Malt extract	Morga, Switzerland
Agar Agar	Morga, Switzerland
Yeast Extract	Thermo Fisher Scientific, Germany

### Maintenance of fungal cultures

Cultures were maintained in vented 90 mm-diameter Petri dishes (VWR, USA), sealed with Parafilm (Bemis Company Inc., USA) by incubation in the dark at $30\,\,^{\circ }\text {C}$ on a defined standard malt agar substrate (SMA), containing 2 wt% malt extract, 2 wt% agar, and 0.2 wt% yeast extract and transferred to a new plate weekly. The SMA substrate was adjusted to higher and lower concentrations of malt extract: Substrates with 5 wt% malt extract (high malt agar, HMA) and substrates with 0.4 wt% malt extract (low malt agar, LMA). All incubation events in this study were run in the dark at $30\,\,^{\circ }\text {C}$ and 80% relative humidity.

### Screening of species

Prior to tackling the nutrient concentration study, a thorough screening of 18 wood fungal species was performed. The preselection of fungal species was based on a literature review (see Supporting Information Table S1). Mycelial morphology was considered, as well as the kinetics of mycelial growth to select fast- and dense-growing species for continued experiments. For the latter, the radial growth rate was measured, which is a valuable tool for assessing the overall growth vigor of fungi^42–44^. To do so, the various species were inoculated on SMA, incubated and their radial growth was measured every 24 h for 7 days. To compare fungal morphology, macroscopic images were taken with a digital single-lens reflex camera (Nikon D800E, AF-S 24-85/3.5-4.5G, Japan) after 7 days. The white balance adjustment (4550 K) of the camera was performed to ensure accurate and consistent color representation in the captured images.

To study the growth kinetics depending on nutrient availability, selected fungal species were inoculated on SMA, HMA, and LMA and measured via the methods described in the following sections.

### Radial growth kinetics of Mycelium

The linear expansion was traced daily until plates were fully colonized (6–7 days). Petri dishes were inoculated in the middle with 5 mm $\emptyset$ discs of mycelium from the edge of 3–5 day old growing colonies. For each day of growth, expansion was measured radially from the point of inoculation, taking 4 measurements per day over a cross drawn on the plate. These values were then averaged for each day and the overall growth speed of the species for each substrate was calculated and reproduced as a graphic using Origin Pro 2021 9.8.0.200 (OriginLab Corporation, USA). For each condition, 3–6 biological replicates were considered.

### Spectrophotometry

Mycelium network density differences when grown on LMA, SMA, and HMA substrates were investigated via spectrophotometry. All experiments were performed in 6-well plates with each well containing 2000 $\upmu$L of solid medium with the appropriate supplements and respective concentrations of malt extract. Plates were inoculated in the middle of the wells with 5 mm $\emptyset$ discs of mycelium and incubated over 7 days. In addition to the mycelium samples, controls containing the same volumes of the respective growth media without mycelium were also included. Every 24 h over 7 days, absorbance was detected and quantified at 595 nm in a Spark Microplate Reader (Tecan, Switzerland).

Data were collected and processed using the software and exported to Origin for additional evaluation and generating graphs. The results are shown as averages of at least three biological replicates. Growth curves were generated by plotting the average absorbance values from center to edge in one well as the growth distance. Furthermore, the absorbance of the mycelial network within one well was quantified by integrating growth curves after 7 days. To allow for better comparison, the obtained absorbance values after 7 days were normalized by subtracting the values from day 0 (controls including the inoculum). The plotted absorbance values over growth distance shown in Supplementary Information are not normalized to different media, however, control values of various media are incorporated into the graphs. Notably, by conducting a visual examination of samples as well as delving into the literature, we are confident that the influence of pigments potentially produced by mycelium on absorbance measurement at 595 nm can be discounted.

### Fungal density via mat weight estimation

The weight of the mycelium mat after two weeks of growth on liquid medium was assessed. Each species was inoculated on LMA, SMA, and HMA medium in 90 mm Petri dishes. Three replicates of each combination of substrate and fungal species were measured.

For inoculation, liquid media was prepared and autoclaved. After cooling the substrate to $60^{\circ }\text {C}$ in a water bath, 200 mL was transferred to an autoclaved blender cup. One-quarter of the overgrown fungal plate (previously grown on SMA for 5 days in the dark at 30°C and 80% RH) was transferred from the plate into the blender cup using a sterile scalpel. The liquid medium was blended with the mycelium for 5 s at maximum speed in three intervals of 5 s with 10 s of pause in between them. Aliquots of 25 mL of the blended mix were transferred into sterile 90 mm Petri dishes using a serological pipette. The Petri dishes were covered with their lids and sealed with Parafilm while being careful not to spill any of the substrates.

After two weeks of incubation, the mycelium was transferred to superstable 50 mL centrifuge (HERMLE Labortechnik GmbH, Germany) tubes and centrifuged for 10 min at 10,000 g. The supernatant was removed and the mycelium was washed by adding 15 mL of water, vortexing, and centrifuging for 10 min at 16,000 g. The supernatant was removed again and the mycelium was filtered through dried, preweighed glass filters to remove the remaining substrate. The filters with the mycelium were dried overnight at $105\,\,^{\circ }\text {C}$ in a drying and heating chamber. The filter and the mycelium were then weighed and the weight of the empty filter was subtracted to obtain the dry mass of the mycelium. A quantitative evaluation for mycelial density was obtained by correlating the obtained weight to the initial substrate volume (25 mL).

### Tensile testing of mycelial mats

Uniaxial tensile tests were conducted via mechanical analysis (MCR 702, Anton Paar, Austria) under controlled shear. The mycelium structures were grown for 14 days on LMA, SMA, and HMA substrates and subsequently dried for 2 h at $60\,\,^{\circ }\text {C}$ to inhibit further growth. Then, the 0.1–0.2 mm thick mycelium mats were gently removed from the substrate and cut into 4 mm wide and 20 mm long strips for mechanical testing. The specimens were deformed at a rate of 2 mm/min and Young’s modulus E was derived from the linear range of the stress-strain curves. The moduli displayed are derived from six measurements taken across three different biological replicates. 

### Descriptive statistics

Statistical analysis was performed within the software Origin Pro 2021 9.8.0.200 (OriginLab Corporation, Northampton, MA, USA). In Fig. 1, the progression of fungal growth is depicted over time (n = 3). In this case, we employed linear regression to facilitate a direct comparison of the daily extension rate. We acknowledge that, given the relatively small sample size, individual data points or ranges might have been more suitable. Nevertheless, for the specific objective of screening species for rapid growth, utilizing linear fits provided the most effective means of comparison.

In Fig. 3a, a comparison of means was performed on the normalized integral absorbance values, and statistical significance was determined using Welch’s t-tests at a significance level of 0.05, considering the absence of assumed equal variance across datasets. Each dataset comprised n = 9 datapoints, and the alpha level was set at 0.05. The p-values resulting from the Welch’s t-test in Fig. 3a were provided in the graph’s description.

### Supplementary Information

Supplementary Information.

## Data Availability

The datasets generated during and/or analyzed during the current study are available from the corresponding author upon reasonable request.
