# Supplementary material for: Impact of malt concentration in solid substrate on mycelial growth and network connectivity in Ganoderma species
Source: Sci Rep. 2023 Nov 29;13:21051. doi: 10.1038/s41598-023-48203-4 (PMC10687231; doi:10.1038/s41598-023-48203-4)
Supplement: Supplementary file 1 — Supplementary Information. [file 41598_2023_48203_MOESM1_ESM.pdf]

Supplementary Information: Impact of Malt Concentration in Solid Substrate on Mycelial Growth and Network Connectivity in Ganoderma Species

Natalie Nussbaum[1,*], Tabea von Wyl[1], Antoni Gandia[2,3], Edwina Romanens[2,+], Patrick Rühs[2,+], and Peter Fischer[1,*]

[1]ETH Zürich, Institute of Food, Nutrition and Health, 8092 Zürich, Switzerland
[2]Planted Foods AG, Kemptpark 32, 8310 Kemptthal, Switzerland
[3]CSIC-UPV, Institute for Plant Molecular and Cell Biology, 46011 Valencia, Spain
[*]natalie.nussbaum@hest.ethz.ch
[*]peter.fischer@hest.ethz.ch
[+]these authors contributed equally to this work

ABSTRACT

With its distinctive material properties, fungal mycelium has surfaced as an innovative material with a diverse array of applications across various industries. This study focuses on how the growth strategies of wood fungi adapt to nutrient availability. The effect of malt extract concentration in the growth medium on radial growth kinetics, morphology, mycelium network connectivity, and mechanical characteristics of mycelium from two *Ganoderma* species were investigated. While the radial growth rate was not significantly influenced by the availability of nutrients within the solid substrate, distinct mycelium network characteristics were observed via spectrophotometry. Increased malt extract contents corresponded to elevated optical density measurements and were visually confirmed by denser mycelium networks in photographic images. Investigating the mechanical characteristics of mycelium cultivated on varying solid substrate concentrations, the Young's modulus exhibited a substantial difference between mycelium grown on 5 wt% malt substrate and samples cultivated on 2 wt% and 0.4 wt% malt substrates. The obtained results represent a new understanding of how malt availability influences mycelial growth of two *Ganoderma* species , a crucial insight for potentially refining mycelium cultivation across diverse applications, including meat alternatives, smart building materials, and alternative leather.

Edibility of *Ganoderma lucidum*

Figure S1 depicts a food product distributed in China, containing *Ganoderma lucidum*. Apart from promising results in preliminary experiments and presence in literature, *Ganoderma lucidum* was included in this study due to its established edibility[1] (Fig. 1)

Screening of fungal species

Various fungal species, chosen based on a literature review[2–8], were screened for promising mycelial growth. Table S1 depicts the fungal species used in this study, including abbreviation and supplier.

Figure S2 shows the preliminary results of growth kinetics of the three fungal species *Ganoderma lucidum*, *Ganoderma sessile* and *Pleurotus ostreatus* var *florida* on SMA over 7 days. From the depicted fits in the growth curves, it is apparent that the fungi exhibit linear growth behavior from days 3 through 7. There is no data point from day 7 for *Ganoderma sessile* since all samples had already fully grown to the edge of the petri dish.

Figure S3 shows the results of additional kinetics measurements of *G.sessile* and *G.lucidum* with substrate containing 2 wt% agar, 0.2 wt% yeast extract, and malt concentrations ranging from 2 wt% to 8 wt%. Extensional growth was measured every 24 hours over 3-4 days. Growth was measured until the mycelium culture had reached the edge of the agar plate, which occurred after 5 days for *G.sessile* and 6 days for *G.lucidum*.

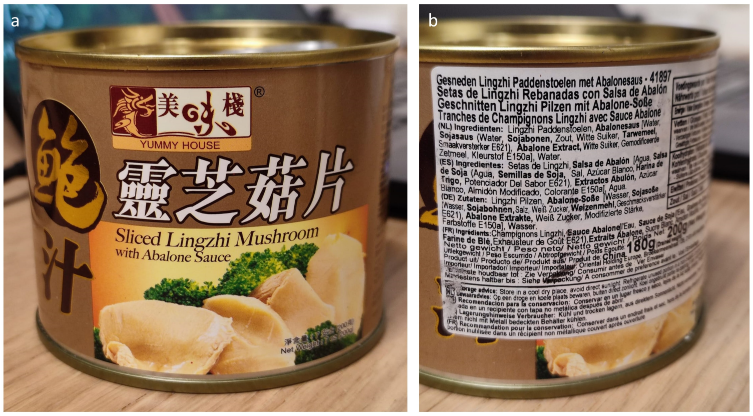

Figure S1. Commercialized canned product of Lingzhi mushroom, i.e. *Ganoderma lucidum* manufactured in China and sold in Europe.

Code	Taxonomic Identification	Phylum	Collection Code	Supplier
AB	*Agaricus bisporus*	Basidiomycota	MG3457	Mycogenetics
ABM	*Agaricus blazai Murill*	Basidiomycota	MG3467	Mycogenetics
AA	*Agrocybe aegerita*	Basidiomycota	MG9301	Mycogenetics
GA	*Ganoderma applanatum*	Basidiomycota	MG11600	Mycogenetics
GL	*Ganoderma lucidum*	Basidiomycota	MG11500	Mycogenetics
GS	*Ganoderma sessile*	Basidiomycota	95-19	MOGU S.r.l.
GF	*Grifola frondosa*	Basidiomycota	MG6110	Mycogenetics
HE	*Hericium erinaceus*	Basidiomycota	MG5500	Mycogenetics
HU	*Hypsizygus ulmarius*	Basidiomycota	MG1505	Mycogenetics
LS	*Laetiporus sulphureus*	Basidiomycota	MG14800	Mycogenetics
MR	*Morchella rufobrunnea*	Ascomycota	MG1601	Mycogenetics
PN	*Pholiota nameko*	Basidiomycota	MG10201	Mycogenetics
PC	*Pleutous citrinopileatus*	Basidiomycota	MG1205	Mycogenetics
PE	*Pleurotus eryngii*	Basidiomycota	MG1105	Mycogenetics
PO	*Pleurotus ostreatus*	Basidiomycota	MG1005	Mycogenetics
POFl	*Pleurotus ostreatus var Florida*	Basidiomycota	MG1015	Mycogenetics
PP	*Pleurotus pulmonarius*	Basidiomycota	MG1305	Mycogenetics

Table S1. Fungal species used in this study including abbreviation and supplier.

Optical Density Measurement

Figure S4 shows representative heatmaps of absorbance measurements, where the signal's dispersion is quantified over one well. At each of the 144 locations, which were organized as a grid of 12 x 12 squares, the individual absorbance values within one well were quantified. The signal is evenly distributed, with lower values near the well borders—which correspond to the center's highly dense mycelium inoculum—and decreasing density near the walls, which represents the expansion of the mycelium over time. Figure S5 shows the average (n=6) OD values of the wells inoculated with *Ganoderma lucidum* (a) and *Ganoderma sessile* (b), measured every 24 hours in-between incubation at 30°C and 80 % RH. The x-axis displays the growth distance [cm] by plotting lines of successive data points from the center (inoculation point) to the edge of the well. Hence, it is important to note that the data points at 1.4 mm growth distance refer to the mycelium inoculation point, which naturally displays a relatively high density from day 0 on. For the quantification of mycelium density in Fig. 2, the integrals of the OD curves after 7 days were compared.

Mechanical analysis

As mentioned in the main text, there are many factors during the preparation for the mechanical analysis of mycelium materials that can influence the resulting characterization. The process phases are summarized in Fig. S6 along with the variables that are impacted by each step. First, the phenotype of fungi used as well as the substrate composition affect mycelium water

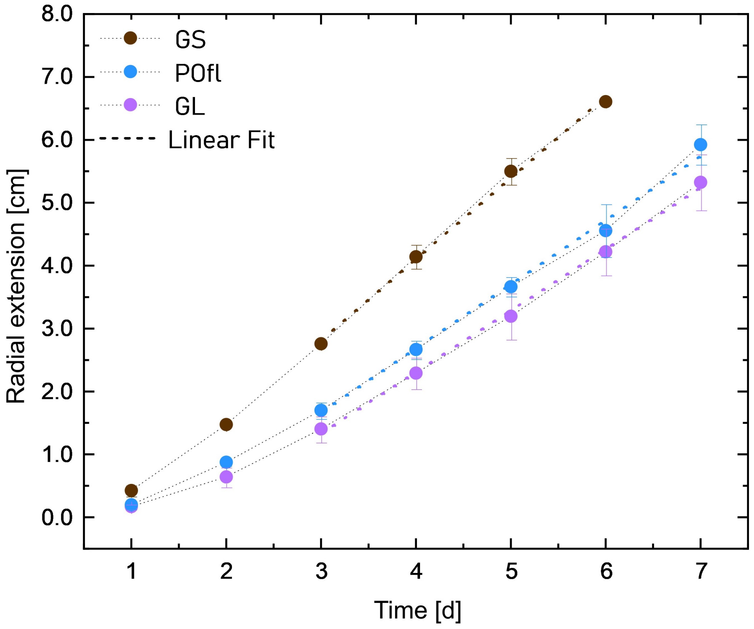

Figure S2. Growth kinetics of *Ganoderma lucidum* (GL), *Ganoderma sessile* (GS) and *Pleurotus ostreatus* var *florida* (POFl). Note: Linear fits with adjusted R squares > 0.99 were considered.

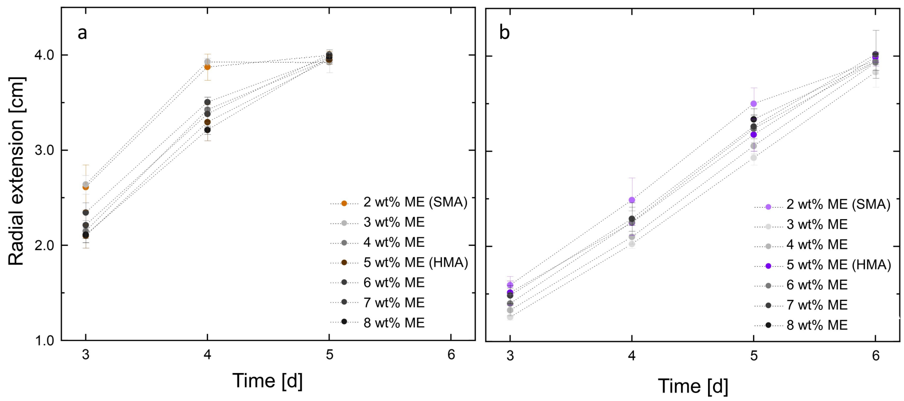

Figure S3. Growth kinetics of a) *G.sessile* and b) *G.lucidum* on agar media with malt concentrations from 2 wt% to 8 wt%.

uptake, growth speed, density, and composition[9,10]. Next, the inoculation condition (inoculum density) has an influence on growth rate and yield[11]. Further, the time of incubation as well as the accuracy of growth conditions account for the mycelium yield. In particular, it can not be discounted that the removal of the mycelium from the substrate has an impact on mycelium structure. Further, proper storage of mycelium samples pre-measuring is needed to avoid mycelium drying out. In addition, pure mycelium samples are very fragile and must be handled with great care when mounting into the tensile geometry to avoid breakage of material. Similar issues occurred in earlier research using tensile tests to study mycelium materials[12,13]. Finally, as shown in Fig. S7, various post-processing processes of the mycelium samples determine numerous attributes, such as water absorption and network density, impacting its mechanical properties[9,10,12,13].

To facilitate data comparison, in figure S7, we compare moduli of pure mycelial materials, while neglecting data on mycelium-composite specimens. The sample preparation for mechanical assessment in this study was based on a study by Haneef et al. (2017)[9]. The measured elastic moduli of the two chosen *Ganoderma* strains are in the same size range (10^{-1} - 10^{1} MPa) as in the reported study[9].

Another technique of sample preparation described in the literature involves hydrolyzing the obtained mycelium mats before mechanical measurement. Obtained values using this sample preparation are slightly higher (10^{0} - 10^{2} MPa). However, it is important to keep in mind, that the water uptake, which affects the final Young's modulus, can itself vary depending on the characteristics of the substrate[9,10]. Further, mycelium mats have been pressed for assessment after drying (green), which

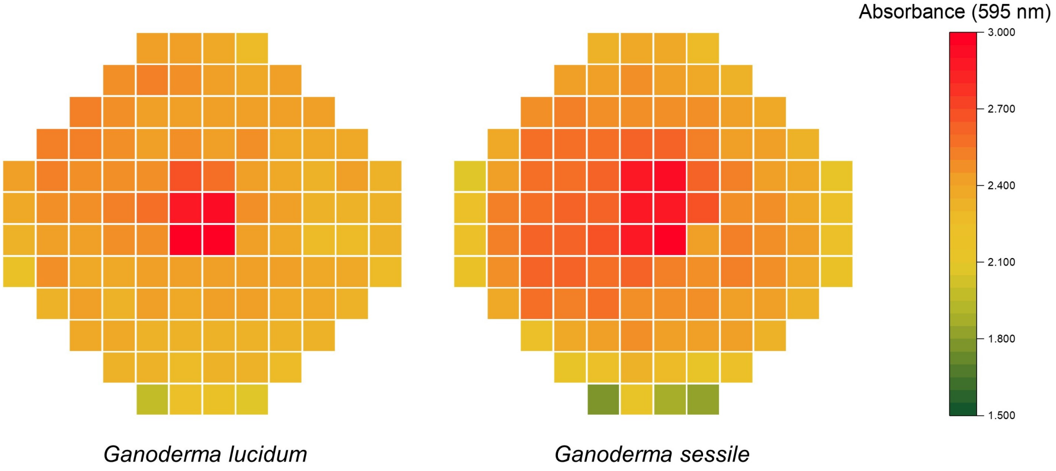

Figure S4. Optical densities of *Ganoderma lucidum* and *Ganoderma sessile* mycelium cultures measured in 12 x 12 square per well at an absorbance of 595 nm.

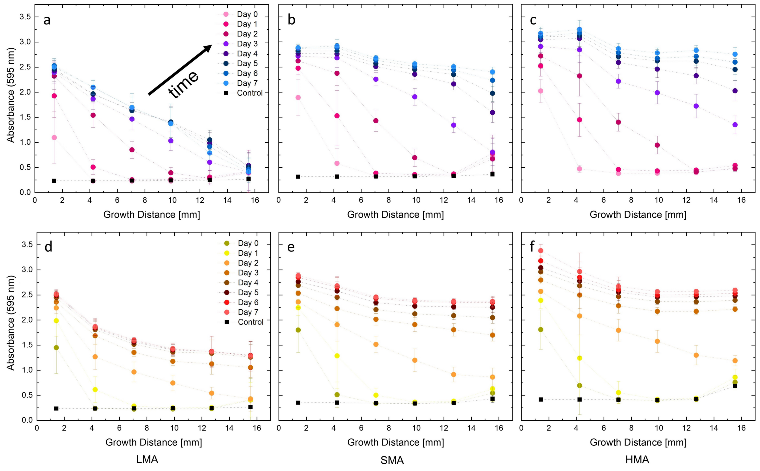

Figure S5. Effect of nutritional conditions on growth speed and density shown via absorbance. Absorbance values at 595 nm of *Ganoderma lucidum* on LMA (a), SMA (b), and HMA (c); similarly, for *Ganoderma sessile* on LMA (d), SMA (e), and HMA (f).

resulted in elastic moduli of a similar size range as hydrolysis. Pressing of mycelium material results in increased material density, which improves the strength of the material[14]. Finally, some measurements were done with mycelium samples that had been layered before drying, which resulted in higher material density and thus, higher elastic moduli.

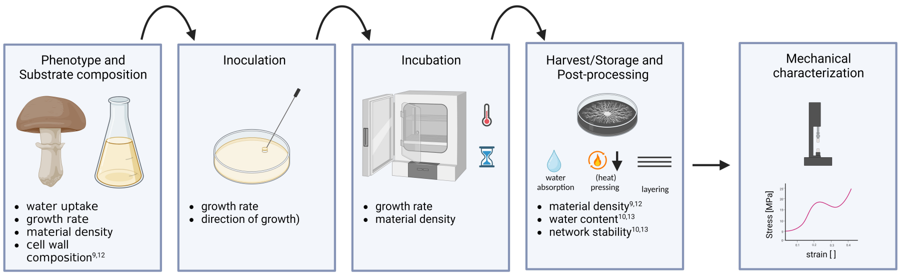

Figure S6. Process flow chart showing the processing steps of mycelium sample preparation for mechanical characterization including a list of affected parameters below[9,10,12,13]. Created with BioRender.com

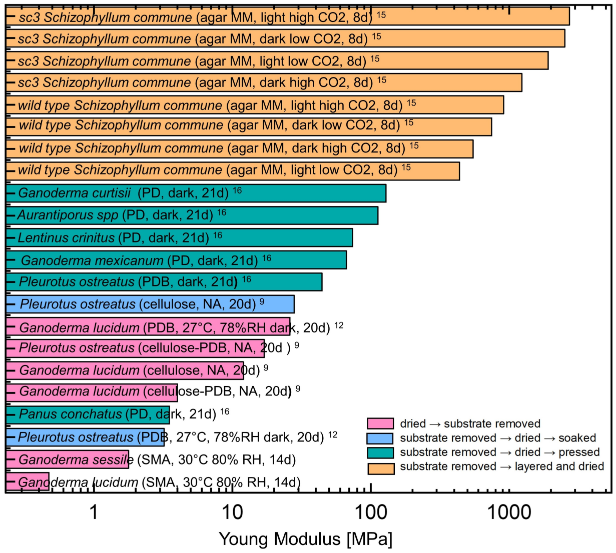

Figure S7. Young's modulus (MPa) of mycelium materials (data not normalized to density). The first two data points are own data, the others from literature[9,12,15,16].

References

1. Ahmad, R. *et al.* Ganoderma lucidum (Reishi) an edible mushroom; a comprehensive and critical review of its nutritional, cosmeceutical, mycochemical, pharmacological, clinical, and toxicological properties. *Phytother. Res.* **35**, 6030–6062, DOI: 10.1002/ptr.7215 (2021).
2. Stamets, P. & Chilton, J. S. *The Mushroom Cultivator: A Practical Guide to Growing Mushrooms at Home* (Ten Speed Press, Berkeley, 1993), 3 edn.
3. Jo, W.-S. *et al.* Culture Conditions for the Mycelial Growth of Ganoderma applanatum . *Mycobiology* **37**, 94, DOI: 10.4489/myco.2009.37.2.094 (2009).
4. Moore, D. & Chiu, S. W. Fungal products as food. *Fungal Divers.* 1–17 (2001).
5. Ma, Y., Guan, C. Y. & Meng, X. J. Biological characteristics for mycelial growth of Agaricus bisporus. *Appl. Mech. Mater.* **508**, 297–302, DOI: 10.4028/www.scientific.net/AMM.508.297 (2014).
6. Luangharn, T., Karunarathna, S. C., Hyde, K. D. & Chukeatirote, E. Optimal conditions of mycelia growth of Laetiporus sulphureus sensu lato. *Mycology* **5**, 221–227, DOI: 10.1080/21501203.2014.957361 (2014).
7. Zied, D. C., Pardo-Giménez, A., de Oliveira, G. A., Carrasco, J. & Zeraik, M. L. Study of Waste Products as Supplements in the Production and Quality of Pleurotus ostreatus var. Florida. *Indian J. Microbiol.* **59**, 328–335, DOI: 10.1007/s12088-019-00805-1 (2019).
8. Viceconte, F. R. *et al.* Ganoderma sessile is a fast polysaccharide producer among Ganoderma species. *Mycologia* **113**, 513–524, DOI: 10.1080/00275514.2020.1870255 (2021).

9. Haneef, M. *et al.* Advanced Materials from Fungal Mycelium: Fabrication and Tuning of Physical Properties. *Sci. Reports* **7**, 41292, DOI: 10.1038/srep41292 (2017).

10. Elsacker, E. *et al.* A comprehensive framework for the production of mycelium-based lignocellulosic composites. *Sci. Total. Environ.* **725**, 138431, DOI: 10.1016/j.scitotenv.2020.138431 (2020).

11. Jones, M., Huynh, T., Dekiwadia, C., Daver, F. & John, S. Mycelium composites: A Review of Engineering Characteristics and Growth Kinetics. *J. Bionanoscience* **11**, 241–257, DOI: 10.1166/jbns.2017.1440 (2017).

12. Antinori, M. E. *et al.* Advanced mycelium materials as potential self-growing biomedical scaffolds. *Sci. Reports* **11**, 1–14, DOI: 10.1038/s41598-021-91572-x (2021).

13. Bucinell, R. B., Keever, R. & Tudryn, G. A Novel Tensile Specimen Configuration for the Characterization of Bulk Mycelium Biopolymer. *Exp. Tech.* **44**, 249–258, DOI: 10.1007/s40799-019-00348-6 (2020).

14. Appels, F. V. & Wösten, H. A. Mycelium Materials. In *Encyclopedia of Mycology*, vol. 2, 710–718, DOI: 10.1016/B978-0-12-809633-8.21131-X (Elsevier, 2021).

15. Appels, F. V. W. *et al.* Hydrophobin gene deletion and environmental growth conditions impact mechanical properties of mycelium by affecting the density of the material. *Sci. Reports* **8**, 4703, DOI: 10.1038/s41598-018-23171-2 (2018).

16. César, E. *et al.* Characterization and Physical Properties of Mycelium Films Obtained from Wild Fungi: Natural Materials for Potential Biotechnological Applications. *J. Polym. Environ.* **29**, 4098–4105, DOI: 10.1007/s10924-021-02178-3 (2021).

List of Figures

S1 Commercialized canned product of Lingzhi mushroom, i.e. *Ganoderma lucidum* manufactured in China and sold in Europe. 2
S2 Growth kinetics of *Ganoderma lucidum* (GL), *Ganoderma sessile* (GS) and *Pleurotus ostreatus* var *florida* (POFl). Note: Linear fits with adjusted R squares > 0.99 were considered. 3
S3 Growth kinetics of a) *G.sessile* and b) *G.lucidum* on agar media with malt concentrations from 2 wt% to 8 wt%. . 3
S4 Optical densities of *Ganoderma lucidum* and *Ganoderma sessile* mycelium cultures measured in 12 x 12 square per well at an absorbance of 595 nm. 4
S5 Effect of nutritional conditions on growth speed and density shown via absorbance. Absorbance values at 595 nm of *Ganoderma lucidum* on LMA (a), SMA (b), and HMA (c); similarly, for *Ganoderma sessile* on LMA (d), SMA (e), and HMA (f). 4
S6 Process flow chart showing the processing steps of mycelium sample preparation for mechanical characterization including a list of affected parameters below[9,10,12,13]. Created with BioRender.com 5
S7 Young's modulus (MPa) of mycelium materials (data not normalized to density). The first two data points are own data, the others from literature[9,12,15,16]. 5

List of Tables

S1 Fungal species used in this study including abbreviation and supplier. 2
